# Density–Nematic Coupling in Isotropic Solution of DNA: Multiscale Model

**DOI:** 10.1002/marc.202400382

**Published:** 2024-08-09

**Authors:** Daniel Svenšek, Jaka Sočan, Matej Praprotnik

**Affiliations:** ^1^ Laboratory for Molecular Modeling National Institute of Chemistry Ljubljana SI‐1001 Slovenia; ^2^ Department of Physics Faculty of Mathematics and Physics University of Ljubljana Ljubljana SI‐1000 Slovenia

**Keywords:** DNA, density–nematic coupling, isotropic phase, linear polymers

## Abstract

Monte Carlo simulations of isotropic solutions of double‐stranded DNA (deoxyribonucleic acid) are performed using the well‐established oxDNA model. By comparing the fluctuation amplitudes with theoretical predictions, the parameters of a generic macroscopic model of an isotropic linear polymer solution/melt are determined. A multiscale continuum field model is thus obtained, corresponding to the full specificity of the isotropic phase of double‐stranded DNA in the usual B‐form as perceived at the macroscopic level. Present research is particularly focused on the coupling between spatial concentration/density variations of the polymer and the emerging nematic orientation order of the chains. This rather unfamiliar, only recently described phenomenon, inherent to linear polymers, is outlined and interpreted. Quantitative predictions are provided for the degree of nematic order induced by concentration gradients in isotropic solutions of double‐stranded DNA.

## Graphical abstract

In linear polymer systems, orientational order of chains is coupled to their density gradients. Using the well‐established oxDNA model, isotropic DNA (deoxiribonucleic acid) solution simulations were performed with varying chain lengths and concentrations, leading to a multiscale continuum model with full specificity of double‐stranded DNA in isotropic phase. The density–nematic coupling strength and the orientational order induced in isotropic DNA solutions are focused.

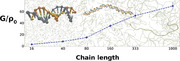

## 1 Introduction

In solutions/melts of linear polymers, gradients of orientational order of polymer chains are coupled to gradients of their concentration/density. This macroscopic consequence of the simple existence of microscopic chains is known as splay–density coupling in nematic polymers, where the splay distortion of the director leads to a density gradient and vice versa.

**Figure 1:**
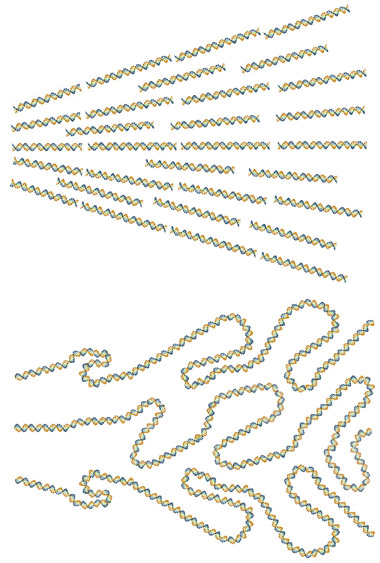
Schematics of splay deformation in a nematic phase. The density of long chains inevitably decreases as the chains spread. Ensembles of shorter chains (top) contain more chain ends that can fill the voids more easily, thus mitigating the decrease in density. Backfolds (bottom) also fill the voids, but reduce orientational ordering unless they are pointlike U turns (hairpins), which are favored in the nematic phase. In this case, they act as chain ends and can fill the voids similar to the top picture.

Traditionally, this phenomenon has been described by the Meyer‐de Gennes continuity constraint^[^ ^1^, ^2^, ^3^, ^4^, ^5^, ^6^, ^7^, ^8^, ^9^, ^10^ ^]^ for the nematic director field n(r) – direction of the polymer chain, valid in the absence of hairpins (sharp, ideally point‐like 180° backfolds of the chain^[^ ^11^ ^]^),

(1) $$\nabla \cdot \left(\rho_{s}n\right)=\rho^{+}-\rho^{-}$$

where $\rho_{s}\left(r\right)$ is the surface number density of polymer chains perforating the plane perpendicular to n and $\rho^{+}\left(r\right)$ and $\rho^{-}\left(r\right)$ are volume densities of the beginnings and endings of the chains, which fill the voids between the chains created by splay, **Figure** **1**, and thus weaken the constraint Equation (1) on $\rho_{s}$ and n.

Nevertheless, hairpin turns of chains are a characteristic feature of nematic order, and despite the awareness of this fact and the fact that Equation (1) seems to be clearly vectorial in nature, it was formally shown only a decade ago that Equation (1) generalizes to a rigorous continuity equation for the polar order vector a(r) instead of the apolar nematic director,^[^ ^12^, ^13^ ^]^

(2) $$\;\nabla \cdot \left(\rho l_{0}a\right)=\rho^{+}-\rho^{-}$$

where the polymer length per unit volume $\rho l_{0}$ is the key quantity. That is, $l_{0}$ is the length of arbitrarily (but of course appropriately) chosen chain segments and ρ is their volume number density, so that the product, the volume density of the polymer length, is a physical fact independent of the choice of the segments. By definition, the polar order vector a describes collective (mesoscopic) polar orientational order of the chains, which can be defined if the chains themselves are polar (directed), i.e., composed of polar monomers.

It was also shown how Equation (1) can be formally adapted so that it can be applied to a nonpolar, uniaxial nematic case with an arbitrary density of hairpins. This is where the so‐called recovered polar order and new effective chain ends with double “strength” corresponding to hairpins come into play (see refs. [13, 14] for the full story). In the limit of large concentration of hairpins,^[^ ^15^ ^]^ however, the continuity equation for the recovered polar order, although still valid, reduces to a trivial identity and therefore loses its meaning.

Nematic orientational order, which is quadrupolar rather than polar, is described by the symmetric traceless nematic order tensor (the quadrupole moment of the orientational distribution function) $Q_{ij}\left(r\right)=\frac{3}{2}\left(\left\langle t_{i}t_{j}\right\rangle -\frac{1}{3}\delta_{ij}\right)$, where, in the case of linear polymers, the averaging ⟨⟩ is over unit vectors t indicating the directions of the chain segments; $\delta_{ij}$ is the Kronecker delta. The director n appearing in Equation (1) is then the principal axis of *Q*_*ij*_. A rigorous conservation law, analogous to Equation (2) for polar order, was derived for quadrupolar (nematic) order,^[^ ^16^ ^]^

(3) $$\partial_{j}\left[\rho l_{0}\left(Q_{ij}+\frac{1}{2}\delta_{ij}\right)\right]=\frac{3}{2}g_{i}+\frac{3}{2}\rho l_{0}k_{i}$$

where the volume density g(r) of chain end directions t, defined as pointing inward, and the volume density $\left(\rho l_{0}k\right)\left(r\right)$ of deflections of consecutive segments play the role of the sources in this continuity equation; k(r) is the local average chain curvature vector. The average chain curvature vector source reflects the effect of general chain folds (including hairpins as their special case), which can fill the voids created by splay in a similar way as the chain ends do, Figure 1 (bottom). The stiffer and longer the chains, the more expensive are the sources and the stronger is the coupling Equation (3) between gradients of $Q_{ij}$ and ρ. Analogous to the polar case Equation (2), the tensorial conservation law Equation (3) is an exact macroscopic implication of the simple existence of the microscopic polymer chains.^[^ ^17^ ^]^

Somewhat unexpectedly, however, Equation (3) is relevant not only for nematic linear polymers, but also for isotropic linear polymers, whether or not they exhibit an isotropic–nematic transition. That is, it applies to any fluid linear polymer (i.e., solution or melt), e.g., synthetic polymers such as polyethylene, polyvinyls, polyamides, polyesters, polystyrene, polycarbonates, etc., and also to a solution of deoxyribonucleic/ribonucleic acid (DNA/RNA) and other linear biopolymers. Why this relevance? Unlike polar order fluctuations, variations of nematic order $\delta Q_{ij}$ are generally coupled in the lowest order to variations of polymer density/concentration δρ even in an orientationally disordered, isotropic phase with $\rho =\rho_{0}$ and $Q_{ij}=0$ in equilibrium, as follows from linearization of Equation (3):

(4) $$\rho_{0}l_{0}\;\partial_{j}\delta Q_{ij}+\frac{1}{2}l_{0}\;\partial_{i}\delta \rho =\frac{3}{2}\delta g_{i}+\frac{3}{2}\rho_{0}l_{0}\;\delta k_{i}$$

This applies to thermal fluctuations as well as to arbitrary, e.g., externally imposed variations δρ(r) and $\delta Q_{ij}\left(r\right)$. Of course, there must exist a microscopic object that can be oriented in the first place (in a fluid of spherical particles, for example, this is not possible), but in polymers such an object is always the polymer chain itself (i.e., even if it consisted of spherical monomers). Therefore, for example, an externally imposed concentration gradient of DNA (or other linear polymer) will induce nematic ordering via the coupling Equation (4) and thereby also optical anisotropy (birefringence) in the otherwise isotropic solution, as has already been shown for the case of a generic worm‐like polymer melt.^[^ ^16^ ^]^

Such osmotic‐stress‐induced birefringence is similar to shear flow‐induced birefringence in fluid polymers and to the stress‐optic law in elastic solid dielectric materials (a direct coupling between strain and dielectric tensors). However, the key difference is that osmotic‐stress‐induced birefringence occurs already in a static liquid where no strain or strain rate tensor exists to couple with the dielectric tensor. Instead, the coupling, which arises from the microscopic polymer chain connectivity, occurs via the concentration gradient as expressed by the tensorial continuity equation Equation (3).

To the best of our knowledge, birefringence induced by polymer concentration gradients has not yet been evidenced in the literature, but the basic arguments presented above suggest that it should be a very persistent, geometrically enforced phenomenon. To theoretically describe this interesting macroscopic mechanism and other phenomena at the macroscopic level, a macroscopic model of the specific polymer is required, which, in order to achieve specificity, must be based on the microscopic information of the concrete system. Hierarchical multiscale modelling has so far proven to efficiently characterize phenomena, which need to be addressed at various levels of complexity,^[^ ^18^ ^]^ such as surface catalyst optimization,^[^ ^19^ ^]^ composite material architecture determination^[^ ^20^ ^]^ and prediction of multi‐principal alloy behavior under stress.^[^ ^21^ ^]^ Here, we employ a multiscale solution that involves a theoretical description of our phenomena at the macroscopic level in conjunction with a micro‐ to mesoscopic description of our polymer of interest. We set up a minimal macroscopic continuum model for a generic isotropic linear polymer solution or melt and determine its parameters corresponding specifically to the isotropic solution of double‐stranded (ds)DNA in B‐form at various chain lengths and concentrations, as simulated with the well‐established oxDNA model.^[^ ^22^ ^]^ The B‐form is the most common isoform of dsDNA, where the planes of the nucleic acid bases are nearly perpendicular to the helical axis. It is the predominant form in biological systems and in vitro aqueous solutions at (quasi) physiological conditions.

## 2 Multiscale Model

Following,^[^ ^16^ ^]^ we use a minimal macroscopic free‐energy density model of an isotropic linear polymer solution or melt that is capable of describing variations of polymer density, nematic orientational order of chains, and coupling between the two:

(5) $$\begin{array}{ccc} f & = & \frac{1}{2}B{\left(\frac{\delta \rho }{\rho_{0}}\right)}^{2}+\frac{1}{2}A{\left(\delta Q_{ij}\right)}^{2}+\frac{1}{2}L{\left(\partial_{k}\delta Q_{ij}\right)}^{2}\\ & & +\;\frac{1}{2}G{\left(\frac{2}{3}\rho_{0}l_{0}\right)}^{2}{\left[\partial_{j}\delta Q_{ij}+\frac{1}{2}\partial_{i}\left(\frac{\delta \rho }{\rho_{0}}\right)\right]}^{2} \end{array}$$

where the variables are the variation of the relative number density of the segments $\delta \tilde{\rho }=\delta \rho /\rho_{0}$, $\rho_{0}$ is its equilibrium value, and the variation of the nematic order tensor $\delta Q_{ij}$, which is zero in equilibrium. The first term describes the cost of density or concentration variations, with B the bulk modulus (in the case of a melt) or the concentration susceptibility (in the case of a solution). The second term captures the cost of nonzero nematic order, which is disfavored in the isotropic system, A is the “stiffness” of the nematic order, i.e., the nematic order modulus. The third term (nematic elastic term) penalizes the gradients of the nematic order. In contrast to the nematic phase, where there are several elastic terms, three by default corresponding to splay, twist and bend distortion modes of the nematic director, there is only one elastic term in the isotropic system. This is due to the fact that there is no preferred direction in equilibrium and thus no distinction between different distortion modes of nematic order is possible. The last term describes the density–nematic coupling. It is enforced by a quadratic potential penalizing the sources of the tensorial conservation law, i.e., the right‐hand side of Equation (4) expressed by its left‐hand side containing the variables; $G{\left(\frac{2}{3}\rho_{0}l_{0}\right)}^{2}\equiv \tilde{G}$ is the strength of the coupling.

Once such a model is established, it is useful in many ways. It provides amplitudes of thermal fluctuations of polymer density and nematic order, the correlation between these two fluctuations, it describes the effects of externally induced density variations or induced nematic order, it can also be supplemented by contributions from static external fields.

**Figure 2:**
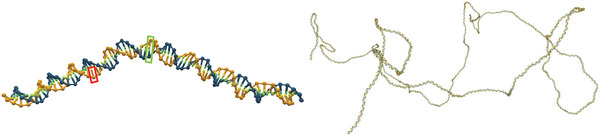
A representation of dsDNA chains of lengths 80 (left) and 5000 (right) base pairs, as modeled by oxDNA. In the oxDNA coarse‐grained force field, each nucleotide (denoted by the red shape) is represented as a rigid body interacting with its surroundings through pairwise interactions, i.e., backbone connectivity and repulsion, as well as nucleobase stacking, repulsion and hydrogen‐bonding. In present work, we treat two hydrogen‐bound nucleotides – the base pair (denoted by the green shape) – as a dsDNA monomer.

The parameters of the model Equation (5), B, A, L, $\tilde{G}$, are unknown and must be determined. A well‐established procedure for this is to compare the amplitudes of long‐wave fluctuations predicted by Equation (5) with the same fluctuations extracted from the actual experimental system. Traditionally, this is done with different types of scattering experiments (X‐ray, neutron, and light scattering).^[^ ^23^, ^24^, ^25^, ^26^, ^27^ ^]^ Nowadays, a complementary and very convenient in silico multiscale strategy is also possible, i.e., performing microscopic simulations and calculating the fluctuation amplitudes directly from numerical data.^[^ ^13^, ^16^, ^28^, ^29^, ^30^, ^31^, ^32^, ^33^, ^34^, ^35^ ^]^ Here, we will use conformational ensembles of dsDNA produced by oxDNA model^[^ ^22^ ^]^ Monte Carlo (MC) simulations, **Figure** **2**.

To obtain the macroscopic fluctuation amplitudes, Equation (5) is expressed in Fourier space,^[^ ^36^ ^]^

(6) $$\begin{array}{ccc} f(q) & = & \frac{1}{2}B|\delta \tilde{\rho }{\left(q\right)|}^{2}+\frac{1}{2}\left(A+Lq^{2}\right){\left|\delta Q_{ij}\left(q\right)\right|}^{2}\\ & & +\;\frac{1}{2}\tilde{G}{\left|q_{j}\delta Q_{ij}\left(q\right)+\frac{1}{2}q_{i}\delta \tilde{\rho }\left(q\right)\right|}^{2} \end{array}$$

where

(7) $$\delta \tilde{\rho }\left(q\right)=\int \;d^{3}r\;\delta \tilde{\rho }\left(r\right)e^{-iq\cdot r},\;\delta Q_{ij}\left(q\right)=\int \;d^{3}r\;\delta Q_{ij}\left(r\right)e^{-iq\cdot r}$$

Thus, the free energy $F=\int \;d^{3}r\;f$ is now a sum over the Fourier modes $F=\left(1/V\right)\sum_{q}f\left(q\right)$, where V is the volume of the system. By equipartition, the energy corresponding to a stand‐alone quadratic contribution $f_{i}\left(q\right)$ of the energy f(q) is $\left\langle f_{i}\left(q\right)\right\rangle /V=k_{B}T/2$, with $k_{B}$ the Boltzmann constant and T the temperature. To determine the fluctuation amplitudes, the quadratic form Equation (6) is diagonalized (see [37] Supporting Information of ref. [16]) and expressed by terms purely quadratic in proper linear combinations of the variables, from which the fluctuation amplitudes follow by equipartition. Since the system is isotropic, we can assume without loss of generality $q=q{\hat{e}}_{z}$ for the fluctuation wave vector, where z is an arbitrarily chosen direction defining the z axis of the coordinate system (the results are the same for any choice of z axis). Axes x and y are then arbitrarily chosen in the plane perpendicular to z and all results at a given q are invariant to rotations of the tensors in the xy plane, in particular $\langle |\delta Q_{xz}{|}^{2}\rangle =\langle |\delta Q_{yz}{|}^{2}\rangle$ and $\langle |\delta Q_{xx}-\delta Q_{yy}{|}^{2}\rangle =4\langle |\delta Q_{xy}{|}^{2}\rangle$. The fluctuation amplitudes are^[^ ^16^ ^]^

(8) $$\begin{array}{ccc} \frac{1}{N_{0}}\langle |\delta Q_{xy}{|}^{2}\rangle & = & \frac{k_{B}T}{2}\frac{1}{\rho_{0}}\frac{1}{A+Lq^{2}} \end{array}$$

(9) $$\begin{array}{ccc} \frac{1}{N_{0}}\langle |\delta Q_{\left\{xz,yz\right\}}{|}^{2}\rangle & = & \frac{k_{B}T}{2}\frac{1}{\rho_{0}}\frac{1}{A+\left(L+\frac{1}{2}\tilde{G}\right)q^{2}} \end{array}$$

(10) $$\begin{array}{ccc} \frac{1}{N_{0}}\langle |\delta Q_{zz}{|}^{2}\rangle & = & \frac{k_{B}T}{2}\frac{4}{\rho_{0}}{\left[3A+\left(3L+\frac{8\tilde{G}B}{4B+\tilde{G}q^{2}}\right)q^{2}\right]}^{-1} \end{array}$$

(11) $$\begin{array}{ccc} \frac{1}{N_{0}}\langle |\delta \tilde{\rho }{|}^{2}\rangle & = & \frac{k_{B}T}{2}\frac{8}{\rho_{0}}{\left[4B+\frac{3\tilde{G}\left(A+Lq^{2}\right)q^{2}}{3A+\left(3L+2\tilde{G}\right)q^{2}}\right]}^{-1} \end{array}$$

(12) $$\begin{array}{ccc} & & \frac{1}{2N_{0}}\left\langle \delta {\tilde{\rho }}^{*}\;\delta Q_{zz}+\delta \tilde{\rho }\;\delta Q_{zz}^{*}\right\rangle \\ & & \;=-\frac{k_{B}T}{2}\frac{1}{\rho_{0}}\frac{8\tilde{G}q^{2}}{12AB+\left[12BL+\left(3A+8B\right)\tilde{G}\right]q^{2}+3\tilde{G}Lq^{4}} \end{array}$$

where $N_{0}\equiv \rho_{0}V$ is the total number of segments in the system. Dividing the fluctuation amplitudes, Equations (8)–(12), by $N_{0}$ is convenient, as in this way they become intensive quantities that do not trivially depend on $N_{0}$. Equation (12) gives the correlation of $\delta \tilde{\rho }\left(q\right)$ and $\delta Q_{zz}\left(q\right)$, i.e., only the component $\delta Q_{zz}$ is directly coupled to density. The fluctuation $\left\langle |\delta Q_{xy}{|}^{2}\right\rangle$ is the only one that does not depend on the coupling strength $\tilde{G}$, i.e., the difference between $\left\langle |\delta Q_{xy}{|}^{2}\right\rangle$ and $\langle |\delta Q_{xz}{|}^{2}\rangle =\langle |\delta Q_{yz}{|}^{2}\rangle$ is a clear signature of the density–nematic coupling in the isotropic phase! Note that $\delta Q_{zz}=-\left(\delta Q_{xx}+\delta Q_{yy}\right)$ because of the tracelessness of $Q_{ij}$.

In Equations (8)–(12), all amplitudes $\delta Q_{xy}$, $\delta Q_{xz}$, $\delta Q_{yz}$, $\delta Q_{zz}$, and $\delta \tilde{\rho }$ of originally dimensionless quantities are Fourier components Equation (7) and therefore have dimensions of V. In microscopic simulations, on the other hand, the Fourier transform is not performed by volume integration of continuous fields, but by summation over discrete polymer segments, so that the transformed quantities remain dimensionless. To allow direct comparison of the fluctuation amplitudes, we multiply the Equations (8)–(12) by $\rho_{0}^{2}$ so that they express the same dimensionless quantities $\left\langle \right|\rho_{0}\delta Q_{xy}{|}^{2}\rangle /N_{0}$,..., and $\left\langle \right|\rho_{0}\delta \tilde{\rho }{|}^{2}\rangle /N_{0}={\left\langle |\delta \rho \right|}^{2}\rangle /N_{0}$. Moreover, from now on we also use dimensionless units by expressing energy relative to $k_{B}T$ and length relative to $l_{0}$. Specifically, the unit of the parameters B, A is $k_{B}T/l_{0}^{3}$, and the unit of the parameters L, $\tilde{G}$ is $k_{B}T/l_{0}$.

With all that, the form of the macroscopic fluctuation amplitudes Equations (8)–(12), which can be compared directly with values obtained from the oxDNA simulations, is

(13) $$\begin{array}{ccc} \frac{1}{N_{0}}\left\langle \right|\rho_{0}\delta Q_{xy}{|}^{2}\rangle & = & \frac{1}{2}\rho_{0}\frac{1}{A+Lq^{2}} \end{array}$$

(14) $$\begin{array}{ccc} \frac{1}{N_{0}}\left\langle \right|\rho_{0}\delta Q_{\left\{xz,yz\right\}}{|}^{2}\rangle & = & \frac{1}{2}\rho_{0}\frac{1}{A+\left(L+\frac{1}{2}\tilde{G}\right)q^{2}} \end{array}$$

(15) $$\begin{array}{ccc} \frac{1}{N_{0}}\left\langle \right|\rho_{0}\delta Q_{zz}{|}^{2}\rangle & = & 2\rho_{0}{\left[3A+\left(3L+\frac{8\tilde{G}B}{4B+\tilde{G}q^{2}}\right)q^{2}\right]}^{-1} \end{array}$$

(16) $$\begin{array}{ccc} \frac{1}{N_{0}}{\left\langle |\delta \rho \right|}^{2}\rangle & = & 4\rho_{0}{\left[4B+\frac{3\tilde{G}\left(A+Lq^{2}\right)q^{2}}{3A+\left(3L+2\tilde{G}\right)q^{2}}\right]}^{-1} \end{array}$$

(17) $$\begin{array}{ccc} & & \frac{1}{2N_{0}}\left\langle \delta \rho^{*}\;\rho_{0}\delta Q_{zz}+\delta \rho \;\rho_{0}\delta Q_{zz}^{*}\right\rangle \\ & & \;=-4\rho_{0}\frac{\tilde{G}q^{2}}{12AB+\left[12BL+\left(3A+8B\right)\tilde{G}\right]q^{2}+3\tilde{G}Lq^{4}} \end{array}$$

## 3 Comparison of Fluctuations, Extracted Model Parameters

**Figure 3:**
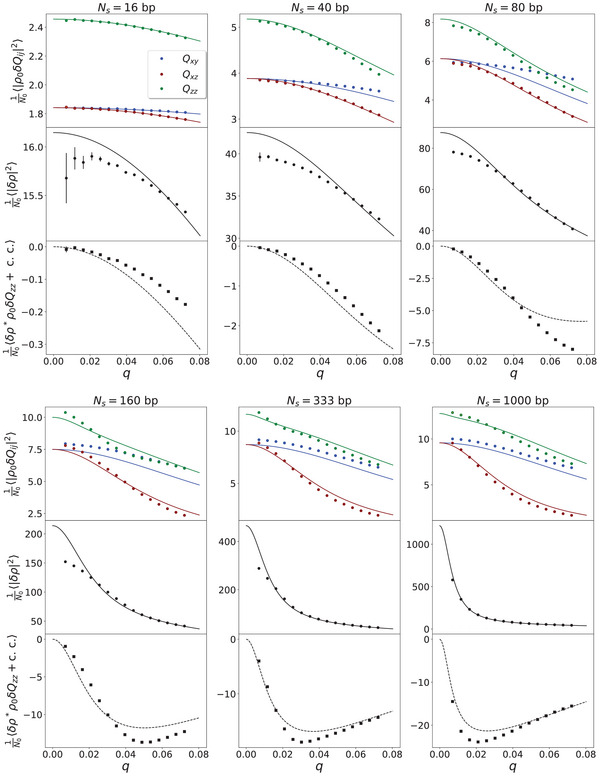
Fluctuation amplitudes and fits to Equations (13)–(16) for different chain lengths $N_{s}$ and base pair density $\rho_{0}=5\rho_{00}\;(\approx 250000$ base pairs). The cross‐correlation curves (dashed) are direct plots of Equation (17) (no fitting). Measured standard deviations are shown by error bars that are too small to be visible except for density fluctuations and cross‐correlations of short chains. The lowest q>0 bin is omitted everywhere because of large statistical errors for short chains.

The full comparison of the macroscopic fluctuation amplitudes, Equations (13)–(17), relevant in the long‐wavelength limit, with the same long‐wavelength fluctuations obtained from the simulations is shown in **Figure** **3** for all six chain lengths and at monomer density $\rho_{0}=5\rho_{00}$ as an example. For a given chain length and monomer density, the fluctuations Equations (13)–(16) are fitted simultaneously to determine the model parameters B, A, L, $\tilde{G}$ for that chain length and monomer density. The cross‐correlation Equation (17) is not fitted but only plotted with the determined parameters. For other simulated densities $\rho_{00}$, $2\rho_{00}$, $10\rho_{00}$, and $20\rho_{00}$, the q‐dependences of all fluctuations and their agreements/discrepancies with theoretical predictions are very similar. The statistical errors are insignificant everywhere, except for the density fluctuation at the lowest q points.

Despite the fact that the system is isotropic (⟨Q⟩=0) and far from any pretransitional orientational effects, the orientation and density fluctuations indeed reveal the fundamental implications of its linear chain structure. The difference between the $Q_{xy}$ and $Q_{xz}$ fluctuations is a direct consequence of the density–nematic coupling and serves as a first indicative measure of the magnitude of the coupling strength $\tilde{G}$ in Equation (14). Already in Figure 3, we can immediately see that it increases systematically with the length of the chains, as expected. According to the discussion after Equation (3) longer chains have fewer ends and consequently the corresponding source g is more expensive, i.e., its fluctuation δg in Equation (4) is smaller and thus the fluctuations of ρ and Q are more strongly coupled.

The *q*‐dependence of the density fluctuation in Figure 3 is also due to this coupling – for $\tilde{G}=0$, ${\left|\delta \rho \right|}^{2}$ would be independent of *q* in this low‐*q* region. We see that it is again much stronger for longer chains. The ultimate manifestation of the density–nematic coupling is, of course, the nonzero cross‐correlation of δρ and $\delta Q_{zz}$ shown in the lower diagrams of Figure 3. Again, it increases systematically with the length of the chains.

Before turning to the details, let us put the results presented in Figure 3 in an overall context. First, they clearly show the presence of the density–nematic coupling. Second, they also show some deviations from the macroscopic model Equation (5) that incorporates this coupling in the simplest manner. We should not forget that the continuity equation Equation (3) is exact and not an approximation. Therefore, it will inevitably manifest itself in some form in the variations of density and orientational order. It is just that the energy cost of the sources of this continuity equation cannot be fully described by the universal quadratic potential of the model Equation (5), and hence the observed discrepancies in Figure 3.

**Figure 4:**
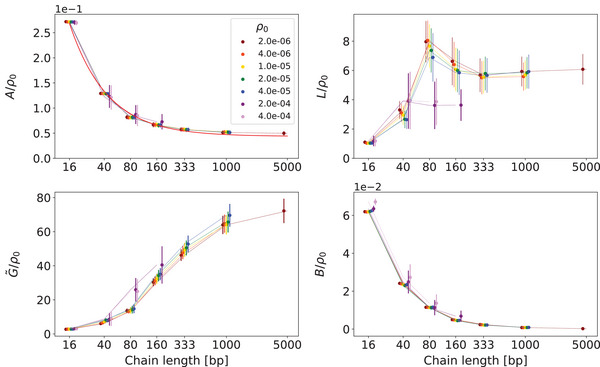
The central result of the simulation: the dependence of the parameters of the model Equation (5) on the polymer chain length $N_{s}$ (note the logarithmic scale) and the density of the monomers $\rho_{0}$. The proportionality of the nematic order stiffness *A* and compressibility *B* to $\rho_{0}$ is exact. For better visibility, the points corresponding to different densities are slightly shifted horizontally. A curve $\approx \;3.60\;(1/N_{s}+1/82.5)$ is fitted to the $A/\rho_{0}$ data.

Our main result is the dependence of the parameters of the model Equation (5) on the polymer chain length $N_{s}$ and the density of the monomers $\rho_{0}$, shown in **Figure** **4** and in **Table** **1**. The expression Equation (5) is a free‐energy density and therefore its parameters are proportional to the monomer density $\rho_{0}$ in first approximation in the absence of other effects. No peculiarities are expected for the compressibility modulus *B* and nematic order stiffness *A*, while this is not so clear in advance for the other two terms with gradients. The plot of the ratios $A/\rho_{0}$, $L/\rho_{0}$, $\tilde{G}/\rho_{0}$, and $B/\rho_{0}$ in Figure 4 shows that for densities $\rho_{00}$ to $20\rho_{00}$ this proportionality is practically exact for all parameters, indicating that the system is essentially dilute (based on crystallographic data,^[^ ^38^ ^]^ at $\rho_{0}=20\rho_{00}$ the solute occupies only about 0.012% of the simulation box volume). There are only minor systematic deviations within the error bars for L and $\tilde{G}$. However, noticeable discrepancies, particularly in $L/\rho_{0}$ and $\tilde{G}/\rho_{0}$, appear for the higher densities of $100\rho_{00}$ and $200\rho_{00}$.

**Table 1:** Parameters of the model Equation (5) at monomer densities $\rho_{00}$, $20\rho_{00}$, and $100\rho_{00}$. The values are the same as plotted in Figure 4, the corresponding units are indicated (these are the $k_{B}T$, $l_{0}$ units defined at the end of Section 2). The listed values of the elastic constant, for example, range from $L=1.20\times 10^{-11}N$ (for $\rho =\rho_{0}=2.5\times 10^{28}m^{-3}\approx 0.050{\mathrm{mg}\;\mathrm{ml}}^{-1}$ and $N_{s}=16$) to $L=4.32\times 10^{-9}N$ (for $\rho =100\rho_{0}\approx 5.0{\mathrm{mg}\;\mathrm{ml}}^{-1}$ and $N_{s}=160$).

	$\rho_{0}$ [$2.5\times 10^{28}m^{-3}$]	16 bp	40 bp	80 bp	160 bp	333 bp	1000 bp	5000 bp
$A/\rho_{0}$ [$4.12\times 10^{-21}J$]	1	0.272	0.129	0.0819	0.0669	0.0574	0.0513	0.0498
	20	0.271	0.129	0.0817	0.0660	0.0569	0.0513	
	100	0.270	0.123	0.0871	0.0727			
$L/\rho_{0}$ [$4.76\times 10^{-40}{Jm}^{2}$]	1	1.01	3.30	7.96	6.62	5.68	5.93	6.08
	20	1.04	2.64	6.89	5.84	5.66	5.92	
	100	1.18	3.91	3.62	3.63			
$\tilde{G}/\rho_{0}$ [$4.76\times 10^{-40}{Jm}^{2}$]	1	2.73	6.08	13.6	30.3	46.3	64.0	72.2
	20	2.84	7.86	14.8	35.1	52.8	69.6	
	100	3.11	8.62	25.9	40.5			
$B/\rho_{0}$ [$4.12\times 10^{-21}J$]	1	0.0620	0.0241	0.0115	0.00499	0.00238	0.000800	0.000222
	20	0.0625	0.0234	0.0114	0.00458	0.00216	0.000869	
	100	0.0635	0.0249	0.0113	0.00683			

**Figure 5:**
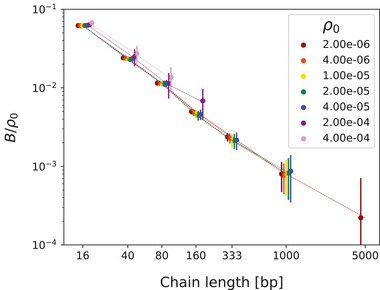
The log–log plot of $B/\rho_{0}$ against chain length $N_{s}$ shows a quasi‐linear dependence. For better visibility, the points corresponding to different densities are slightly shifted horizontally. Excluding the two highest densities, the slope of the line through the points $N_{s}=16,40,80$ is approximately −1.17.

The decreasing dependence of the compressibility modulus *B* on the chain length, Figure 4, is in agreement with the known theoretical result for an ideal polymer chain (entropic compressibility modulus)^[^ ^39^ ^]^, pp. 19–20].^[^ ^40^ ^]^ Namely, in the limiting case of weak confinement, i.e., when the average gyration radius (rms radius) $r_{g}$ of the chain is much smaller than the confining box of volume *V*, the pressure exerted by the ideal chain is simply $p\approx k_{B}T/V$ and the compressibility modulus is $B=\left(1/\rho \right)\partial p/\partial \rho =-V\partial p/\partial V\approx k_{B}T/V\approx p$. That is, in this limit the ideal chain behaves like a single ideal gas particle. For $N_{c}$ independent ideal chains, this means $B\approx p\approx N_{c}k_{B}T/V=\rho_{0}k_{B}T/N_{s}$. Thus, in our dimensionless units the ratio $B/\rho_{0}$, which is shown in Figure 4, would ideally be $B/\rho_{0}\approx 1/N_{s}$, i.e., $\log\left(B/\rho_{0}\right)\approx -\log N_{s}$. The log–log plot in **Figure** **5** indeed shows a quasi‐linear dependence with a slope close to −1. For longer chains, the line rises slightly, which may indicate that $r_{g}$ of these chains is no longer very small compared to the size of the box, and their segments begin to make individual contributions to the pressure. For the two higher densities, $B/\rho_{0}$ is slightly higher and in particular the lines in Figure 5 rise more strongly, which points to the effect of chain's reduced free volume. This volume, rather than the full volume of the box, is now to be compared to the volume $4\pi r_{g}^{3}/3$ occupied by a chain.

Also of entropic origin is the decreasing dependence of the nematic order modulus *A* on the chain length in Figure 4. For a fixed number of segments, stochastic fluctuations of the collective (mesoscopic average) orientational order are smaller for independent segments than for segments bound in a chain. This is simply because the latter have fewer independent orientational degrees of freedom and contribute fewer independent random orientations to the average orientation. As a result, statistical variations of the average orientation are larger and thus a nonzero nematic order is more probable, which translates into a smaller *A*. For longer and hence fewer chains, however, the intra‐chain orientational freedom of the segments begins to dominate, which is larger for more flexible chains. A curve $A\left(N_{s}\right)/\rho_{0}\approx \;3.60\;\left(1/N_{s}+1/82.5\right)$ fits well the $A/\rho_{0}$ data in Figure 4, where the last term is of the order of the inverse persistence length of the chain $\xi_{p}$ in units of the segment length, which will be discussed in more detail in Section 4.

The non‐monotonic behavior of the elastic constant $L/\rho_{0}$, Figure 4, is rather surprising and remains unaccounted for. It occurs in the regime, where the chain length grows larger than the persistence length, i.e., when energetic nematic elasticity transitions to entropic elasticity. We should bear in mind, however, that our system is in the isotropic phase, where one cannot expect a well‐defined nematic elastic constant.

## 4 Density–Nematic Coupling Strength

Our prime focus is on the strength of the density–nematic coupling and its dependence on the polymer length. As confirmed by the $\tilde{G}/\rho_{0}$ plot in Figure 4, the importance of the coupling, i.e., the relative magnitude of $\tilde{G}$ with respect to the other model parameters, does not significantly depend on density. In other words, the coupling is already equally effective in the dilute limit and thus ubiquitous for all solutions of DNA, as well as for linear polymeric liquids in general! Moreover, from Table 1 it can be seen that the coupling strength $\tilde{G}$ is substantially larger than the nematic elastic constant *L*, which will prove to be important in the following. The ratio $\tilde{G}/L$ increases with chain length and already becomes larger than ten for $N_{s}=1000$. Note that the $\tilde{G}$ values given represent the actual “measured” strengths of the density–nematic coupling for DNA solution as simulated by the oxDNA model. This is our main empirical result, which fully quantifies the density–nematic coupling. Before comparing it with a theoretical model in Section 4.1, one of our ancillary interests, let us put it in a practical perspective and estimate what it means for the magnitude of the induced nematic order effect. That is, let us connect spatial variations in polymer concentration with the degree of nematic ordering they induce.

We now assume an externally imposed monomer number density profile δρ depending only on the *z* coordinate for simplicity. A nonhomogeneous concentration profile can, for example, be sustained between reservoirs with different chemical potentials. Inclusions such as droplets, bubbles, or impurities – frequently encountered in experimental samples – also act as spatial modulators of the chemical potential. A wave‐like, periodic or solitonic concentration profile frequently results spontaneously from a spatial instability in a frustrated system, including phase separation. It follows from the model Equation (5) with the non‐linearized conservation law Equation (3) for generality (Supporting Information of ref. [16] Section VI, Equation (49))

(18) $$\begin{array}{ccc} & & \left(L+G^{\prime }\rho^{2}\right)\partial_{z}^{2}\delta Q_{zz}+2G^{\prime }\rho \left(\partial_{z}\delta \rho \right)\partial_{z}\delta Q_{zz}\\ & & \;+\;G^{\prime }\;\left(\delta Q_{zz}+\frac{1}{2}\right)\;\rho \partial_{z}^{2}\delta \rho -A\delta Q_{zz}=0 \end{array}$$

where $G^{\prime }\equiv G{\left(\frac{2}{3}l_{0}\right)}^{2}$. This rather complicated connection between the externally imposed δρ(z) profile and the resulting uniaxial nematic order profile $\delta Q_{zz}\left(z\right)$ can be reduced to extremely simple expressions in the following special limiting cases.

**i)** A small amplitude wave‐like (sinusoidal) modulation of relative monomer density $\delta \tilde{\rho }=\delta \rho /\rho_{0}$ with wave vector $q=q{\hat{e}}_{z}$ results in the explicit wave‐like solution for the induced nematic order,

(19) $$\delta Q_{zz}\left(z\right)=-\frac{1}{2}\frac{\tilde{G}q^{2}}{A+\left(L+\tilde{G}\right)q^{2}}\delta \tilde{\rho }\left(z\right)$$

For long‐wavelength density modulations, i.e., when the wavelength is large with respect to $2\pi \sqrt{(L+\tilde{G})/A}$, which is of the order of the persistence length of the chain, we have $\left(L+\tilde{G}\right)q^{2}\ll A$ and thus only a small induced nematic order $\delta Q_{zz}\approx -\frac{1}{2}\left(\tilde{G}q^{2}/A\right)\;\delta \tilde{\rho }$. Conversely, for short‐wavelength modulations, the induced nematic order is $\delta Q_{zz}\approx -\frac{1}{2}{\left(1+L/\tilde{G}\right)}^{-1}\;\delta \tilde{\rho }$ and is of the same order as the relative density variation. The minus sign indicates prolate (axial) ordering of the polymer chain in regions of decreased concentration and oblate (planar) ordering in regions of increased concentration. Note that a perfect prolate order has $Q_{zz}=1$ and a perfect oblate order has $Q_{zz}=-1/2$.

**ii)** When a general concentration profile varies slowly on the length scale $\sqrt{(\tilde{G}+L)/A}$, we get from Equation (18) in the limit $\delta Q_{zz}\to 0$

(20) $$\partial_{z}^{2}Q_{zz}\left(z\right)\approx -\frac{1}{2}\frac{1}{1+L/\tilde{G}}\;\partial_{z}^{2}\delta \tilde{\rho }\left(z\right)$$

That is, a non‐uniform relative concentration gradient induces a comparably strong spatial variation of the gradient of the uniaxial nematic order.

We see that the ratio $L/\tilde{G}$ is the relevant parameter in both cases. If it is small, as we found to be the case in DNA solution, the induced nematic order is substantial – it is of the same order as the relative variation of polymer concentration.

### 4.1 Comparison With a Theoretical Model for *G*

The observed leading proportionality of $\tilde{G}$ to the monomer density $\rho_{0}$, i.e., the leading $1/\rho_{0}$ dependence of the original parameter *G* of Equation (5), is corroborated by the simplest models of the free‐energy costs of the sources of Equations (3)–(4) developed in ref. [16]. In these models (see Supporting Information of ref. [16] for derivation), the chain ends are treated as independent vectors (ideal gas of dipolar particles) that contribute to g, and the kinks between chain segments as independent contributions to $\rho_{0}l_{0}k$ (recall the definition of both types of sources following Equation (3)). Chain ends give the entropic free‐energy density (Equation (29) of Supporting Information [16]), in the present dimensionless units,

(21) $$\Delta f\left(g\right)=\frac{1}{2}\frac{3}{\rho_{0}^{\pm }}g^{2}=\frac{1}{2}\frac{3N_{s}}{2\rho_{0}}g^{2}$$

where $\rho_{0}^{\pm }=2N_{c}/V=2\rho_{0}/N_{s}$ is the number density of chain ends.^[^ ^41^ ^]^ Kinks between chain segments give the free‐energy density (Equation (32) of Supporting Information [16]), in the present dimensionless units,

(22) $$\Delta f\left(k\right)=\frac{1}{2}\frac{3\varepsilon_{T}}{2\rho_{0}}{\left(\rho_{0}l_{0}k\right)}^{2}$$

coming from the bending energy of the kinks with dimensionless bending stiffness $\varepsilon_{T}\equiv \varepsilon /\left(k_{B}T\right)$ and bending stiffness ε. Note that $\varepsilon_{T}$ is exactly the dimensionless persistence length $\xi_{p}$ of the chain,[^[^ ^42^ ^]^, p. 399] i.e., the persistence length in units of the segment length $l_{0}$.

We see that both quadratic free energies, Equations (21) and (22) are indeed proportional to 1/ρ_0_. However, they are two distinct and practically independent contributions, not a single contribution like the simplistic quadratic penalty potential assumed in the model Equation (5). To get around the inconvenience, we introduce a joint free‐energy cost $\Delta f\left(h\right)\equiv \frac{1}{2}Gh^{2}$ of the combined source $h=g+\rho_{0}l_{0}k$, i.e., a convenient approximation that allows us to live with the single penalty potential of Equation (5). It is obtained by averaging the sum of Equations (21)–(22) over all possible realizations of h with respect to g and k (see Supporting Information of ref. [16] for concept and derivation). The resulting dimensionless strength of the penalty potential is, quite elegantly,^[^ ^16^ ^]^

(23) $$G=\frac{3}{2}\frac{1}{\rho_{0}}{\left(\frac{1}{N_{s}}+\frac{1}{\varepsilon_{T}}\right)}^{-1}$$

For increasing ratio $N_{s}/\varepsilon_{T}$ it shows a crossover from chain‐end‐ to chain‐curvature‐dominated strength, which is at $N_{s}=\varepsilon_{T}$, i.e., when the persistence length equals the length of the chain. For chains much shorter than the persistence length, we have $G\approx \left(3/2\right)N_{s}/\rho_{0}$, which also follows directly from Equation (21). This is the regime of stiff chains, where the strength of the density–nematic coupling increases proportionally with the number of segments $N_{s}$ of the chains. Conversely, for chains much longer than the persistence length, $G\approx \left(3/2\right)\varepsilon_{T}/\rho_{0}$, which in turn can be directly deduced from Equation (22). This is the regime of flexible chains, where the coupling strength is controlled by the ratio of bending stiffness and thermal energy. Comparison with $G\approx \left(3/2\right)N_{s}/\rho_{0}$ of the stiff regime shows that the persistence length $\varepsilon_{T}\equiv N_{s}^{\mathrm{eff}}$ is simply the new effective chain length $N_{s}^{\mathrm{eff}}$, defining the coupling strength in the flexible regime.

To recap, the theoretical coupling strength Equation (23) results from the simplest free‐energy models of independent sources of the tensorial conservation law Equation (3), which are additionally approximated as a single source. Does it bear any resemblance to what we measured? The $\tilde{G}/\rho_{0}$ plot in Figure 4 indeed shows the cross‐over behavior with respect to chain length $N_{s}$. It also shows saturation for large $N_{s}$, which is to be expected according to Equation (23), since in our case the bending stiffness ε of DNA is a fixed parameter and thus its persistence length $\varepsilon_{T}$ at fixed temperature is also fixed. Thus, we can already confirm at least this qualitative agreement.

**Figure 6:**
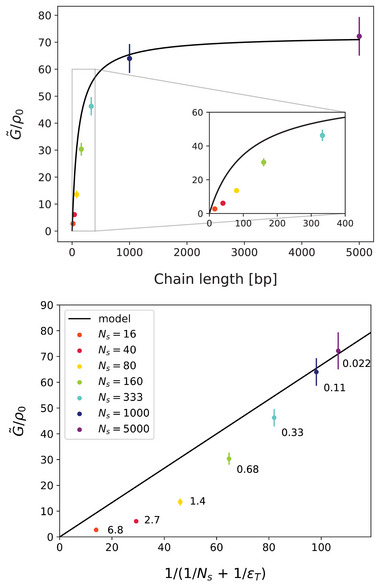
Top: fit of the model Equation (23) with fit parameter $\varepsilon_{T}$ to the 1000 and 5000 bp points of the $\tilde{G}/\rho_{0}$ data from Figure 4; $\rho_{0}=\rho_{00}$. Bottom: the same data plotted against the expression in Equation (23) (the theoretical curve above is now a straight line). For each data point, the value of the ratio $\varepsilon_{T}/N_{s}$ is given.

Next, we try to fit the measured $\tilde{G}/\rho_{0}$ data from Figure 4 with the model Equation (23), i.e., explicitly with

(24) $$\tilde{G}/\rho_{0}=\frac{2}{3}{\left(\frac{1}{N_{s}}+\frac{1}{\varepsilon_{T}}\right)}^{-1}$$

and the only fit parameter $\varepsilon_{T}$. A fit of the $\rho_{0}=\rho_{00}$ data taking into account only points $N_{s}=1000$ and $N_{s}=5000$, shown in **Figure** **6** (top), yields $\varepsilon_{T}\approx 109$, which is a good estimate of the persistence length (i.e., 109 base pairs). Fits with the last four, three, two, and one of the points $N_{s}=\left\{160,333,1000,5000\right\}$ all give similar values in the range $\varepsilon_{T}\approx 102$ to $\varepsilon_{T}\approx 111$. Systematic deviations from the theoretical model are observed for shorter chains. In Figure 6 (bottom), the plot of the same data as a function of the expression Equation (24) with $\varepsilon_{T}=109$ shows more clearly these discrepancies from the theoretical model Equation (24) (now the straight line). In general, the same value on the horizontal axis can be realized by different combinations of $N_{s}$ and $\varepsilon_{T}$. In this particular case, $\varepsilon_{T}$ is fixed and only $N_{s}$ changes – the corresponding ratios $\varepsilon_{T}/N_{s}$ are given with the data points.

**Figure 7:**
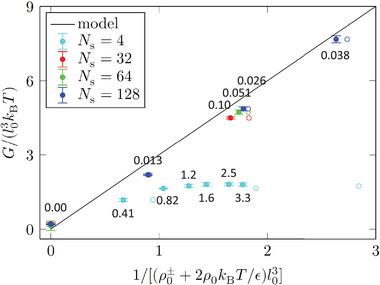
A plot of the dimensionless coupling strength *G* from ref. [16] analogous to Figure 6 (bottom), for a MC‐simulated generic isotropic melt of discrete worm‐like chains within a mesoscopic “soft” model^[^ ^13^, ^33^ ^]^ (solid circles with error bars). The values of the $\varepsilon_{T}/N_{s}$ ratio are again given for all solid circles and can be directly compared with those in Figure 6 (bottom). Empty circles denote abscissae without the $\rho_{0}^{\pm }$ term, i.e., with neglected theoretical contributions from the chain ends (three rightmost $N_{s}=4$ empty circles lie outside the plot and are not shown). In contrast to Figure 6 only the shortest chains come out of the flexible regime. The permission to reproduce and edit the figure was granted by the author.

A worthwhile comparison can be made with an analogous plot in **Figure** **7**, which has been obtained in ref. [16] by MC simulations of a generic isotropic melt of discrete worm‐like chains described by a mesoscopic “soft” model.^[^ ^13^, ^33^ ^]^ This is a very different system, but the density–nematic coupling is universal for linear polymers, as is the form of the model Equation (5). In that case, there is data available for four chain lengths $N_{s}=4,32,64,128$ and bending stiffnesses from $\varepsilon_{T}=0$ to $\varepsilon_{T}=5.0$ for $N_{s}=128$ and up to $\varepsilon_{T}=13$ for $N_{s}=4$, while only $\varepsilon_{T}=0$ and 3.3 for $N_{s}=32$ and 64. In Figure 7, the corresponding ratios $\varepsilon_{T}/N_{s}$ are again given with the data points. This is a valuable complement to our current data in Figure 6, since the different values on the horizontal axis of Figure 7 are realized predominantly by variations of $\varepsilon_{T}$ rater than $N_{s}$. Except for the very short $N_{s}=4$ chains, where the theoretical model breaks down due to the small number of intra‐chain conformational degrees of freedom, the ratios $\varepsilon_{T}/N_{s}$ are small and correspond to the regime of flexible chains. That is, $\tilde{G}$ is controlled predominantly by $\varepsilon_{T}$ and is only weakly affected by $N_{s}$, which is also directly confirmed in Figure 7 by the three closely points for equal $\varepsilon_{T}$ and $N_{s}=32,64,128$ with ratios 0.10,0.051,0.026, respectively. In Figure 6 (bottom), on the other hand, we gradually cross over to the regime of stiff chains characterized by large $\varepsilon_{T}/N_{s}$ ratios. Here, the crossover length (i.e., persistence length, $\varepsilon_{T}$) is much larger than the few segments in the cases of Figure 7, yet the expression Equation (24) already deviates significantly from the measured data (although less severe than the stiffer $N_{s}=4$ chains in Figure 7).

The new data in Figure 6 (bottom) thus indicate that the theoretical model Equations (21)–(24) for the density–nematic coupling strength *G* is inaccurate not only for very short chains with few conformational degress of freedom (segments), but generally also for stiff ($\varepsilon_{T}/N_{s}\ll 1$) and semi‐flexible ($\varepsilon_{T}/N_{s}\approx 1$) chains, regardless of the number of their segments. Nevertheless, this model is useful also in these regimes and incorporates the influence of the chain ends quite well – without it, the curve in Figure 6 (top) would be just a constant, and the data points in Figure 6 (bottom) would all be on the same(!) abscissa (=109). Taking the chain ends into account clearly corrects the prediction of *G* qualitatively in the right direction, and moreover the quantitative inaccuracy for semi‐flexible chains is still below 50%.

## 5 Conclusion

We have set up a multiscale macroscopic model of an isotropic solution of double‐stranded DNA in B‐form that comprises the macroscopic polymer concentration field and nematic orientational order field as variables. By conducting microscopic MC simulations employing oxDNA model we determined the parameters of the macroscopic model for various DNA chain lengths from 16 to 5000 base pairs and concentrations from $0.050\;{\mathrm{mg}\;\mathrm{ml}}^{-1}$ to $10\;{\mathrm{mg}\;\mathrm{ml}}^{-1}$, which is marginally high for the isotropic phase, at a temperature 25°C and an ionic strength [Na^+^] of 1M. A detailed systematic characterization of this multiscale DNA model, including coverage of temperature and ionic strength ranges, will be carried out in a subsequent study in which macroscopic aspects of the double strand decomposition are of prime interest. Allowing such a decomposition and similar decompositions in other multi‐stranded linear polymers, e.g., collagen, requires a generalization of the model for the density–nematic coupling strength.

We found that, to a first approximation, the parameters of the model are simply proportional to the concentration for all concentrations studied, except of the two highest ones. In particular, this means that the coupling between concentration and nematic orientational order, which was our main concern, is already equally effective in the dilute limit and thus inherent to all solutions of DNA (and linear polymer liquids in general), regardless of the concentration. As expected, the coupling strength increases with chain length and saturates for long chains at a value determined by the persistence length.

The results show that for sufficiently long DNA chains, i.e., with a length of at least the persistence length or longer, the density(concentration)–nematic coupling strength $\tilde{G}$ is significantly larger than the elastic constant *L*. Consequently, the induced nematic orientational order is of the same order of magnitude as the relative concentration variation.

To the best of our knowledge, birefringence induced by polymer concentration gradients has not yet been experimentally reported. Nevertheless, due to its fundamental geometrical origin, it should be a very persistent, robust phenomenon, but its large effects may be limited to short length scales supporting large concentration gradients. A large second derivative (curvature) of the concentration field, which is the driving cause of nematic ordering, is typically found in the vicinity of small inclusions of another phase, such as bubbles or droplets. The present work should also serve as motivation for the experimental observation, characterization, and quantification of this interesting phenomenon of birefringence due to nematic order induced by spatial variations in polymer concentration.

In addition to the macroscopic aspects of temperature‐controlled DNA double strand decomposition and other detailed aspects of isotropic double‐stranded DNA solution, our future perspective includes the formulation of its nematic phase analogue, as well as the creation of a variety of multiscale models of other isotropic/nematic linear polymer solutions or melts.

## 6 Methods Section

Simulations were performed at 25°C (298.16K) and an ionic strength $\left[{\mathrm{Na}}^{+}\right]$ of 1M. The salt concentration above the typical physiological concentration range 0.01 – 0.1M allowed us to efficiently simulate extensive systems with up to 1.0 × 10^5^ base pairs due to a shorter Debye screening length cut‐off.

Double‐stranded DNA polymers in B‐form were constructed using the oxDNA^[^ ^22^ ^]^ generation setup, Figure 2, where each nucleotide base pair is represented as a coarse‐grained particle interacting with its environment through backbone connectivity, excluded volume, cross‐chain hydrogen bonding and base stacking potentials.^[^ ^43^ ^]^ The B‐form, as the by far most common conformation of the DNA macromolecule in aqueous solutions, is excellently represented by the oxDNA coarse‐grained implicit solvent model, which enables the exploration of polymer properties in systems comprising 100 000 base pairs or more. For the present purpose, complementary polyguanine and polycytosine chains were simply used, since it had been found that the observed macroscopic properties of the model at the temperature considered did not depend on the specific base pair sequence. Thus, the segments were the guanine‐cytosine base pairs with segment length (our length unit) $l_{0}=0.34\;\mathrm{nm}$.^[^ ^44^ ^]^

Generally, the oxDNA model has been shown^[^ ^22^ ^]^ to offer a good representation of structural and mechanical properties, such as persistence length or response to either interal or external stresses, for various nucleic acid polymers. Moreover, it is particularly well calibrated to reproduce DNA denaturation and hybridization, i.e., dissociation and reassociation of double strand into single strands and vice versa. A separate study is planned to investigate sequence‐dependent macroscopic consequences of double strand dissociation, along with their dependence on temperature and ionic strength.

**Figure 8:**
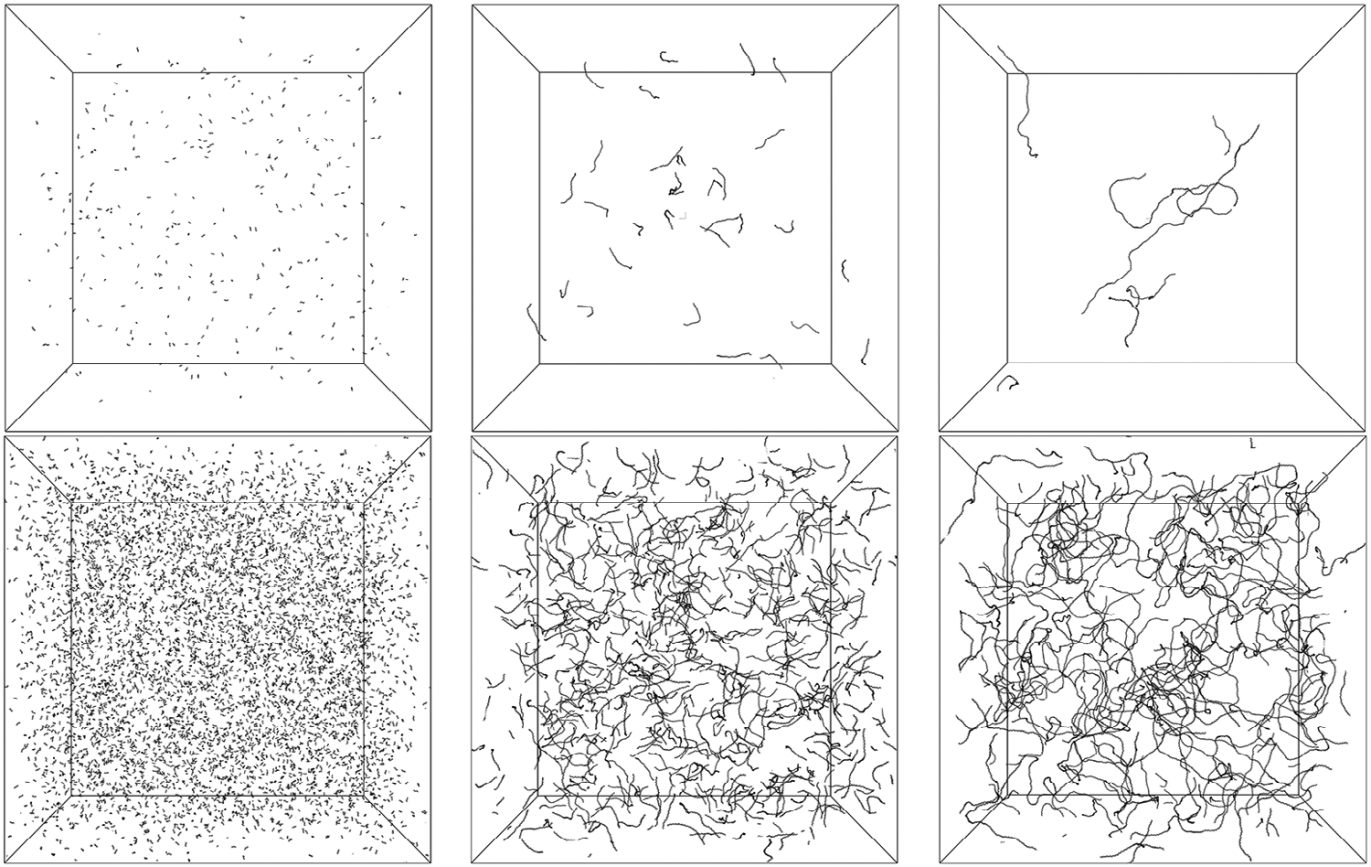
Simulation snapshot examples of systems with 16, 160, and 1000 base pairs per chain and base pair densities $\rho_{00}$ (top) and $20\rho_{00}$ (bottom).

A cubic simulation box with a fixed side of $L_{0}=1365$ was used (NVT conditions) and periodic boundary conditions were applied. As it was essential for the DNA to be in the isotropic phase, its concentration had to be kept below the level at which cholesteric (chiral nematic) liquid crystalline phase (co‐)exists. A systematic array of systems was set up with five different segment (base pair) number densities, multiples 1, 2, 5, 10, and 20 of the base density $\rho_{00}=2.0\times 10^{-6}$ (corresponding to about $0.050\;{\mathrm{mg}\;\mathrm{ml}}^{-1}$), and monodisperse polymer chains of $N_{s}=$ 16, 40, 80, 160, 333, and 1000 base pairs at each density, as illustrated in **Figure** **8**. To keep the chain length strictly monodisperse, $\rho_{00}$ was minimally adjusted as needed by rounding the total number of monomers to the nearest multiple (i.e., integer number of chains $N_{c}$) of the chain length. This density interval is well below the threshold concentration $\approx 10\;{\mathrm{mg}\;\mathrm{ml}}^{-1}$ for the isotropic to cholesteric phase transition of DNA at $[{\mathrm{Na}}^{+}]=0.1\;M$.^[^ ^45^ ^]^ For still higher densities, yet compatible with the isotropic phase, additional systems were set up with $\rho_{0}=100\rho_{00}$, $N_{s}=$ 16, 40, 80, 160 and $\rho_{0}=200\rho_{00}$, $N_{s}=$ 16, 40, 80 in a box with $L_{0}=1365/\sqrt[{3}]{100}$, while checking that the degree of nematic order (the average largest in magnitude eigenvalue of $Q_{ij}$) was below ≈0.01 and the equilibrium phase remained essentially isotropic.

The simulations were performed using the virtual‐move Monte Carlo (VMMC) protocol^[^ ^43^, ^46^ ^]^ – a MC algorithm particularly suited for computational models expressed only by pairwise interactions between particles (such as oxDNA). In such an MC step, clusters of interacting particles are moved together while preserving their internal configuration. Under NVT conditions, the VMMC algorithm performs translational and rotational moves of the clusters. The clustering is also subjected to MC evaluation, so strongly bonded particles are only more likely clustered and moved together, while intra‐chain MC moves remain present.

**Figure 9:**
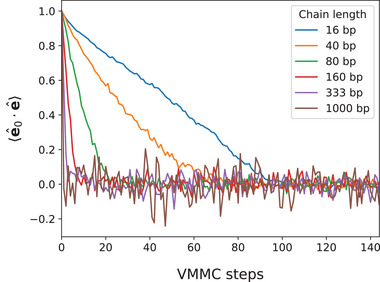
Average projection of the end‐to‐end directions of the chains (unit vectors $\hat{e}$) onto these directions in the initial configuration (unit vectors ${\hat{e}}_{0}$), decreasing with the number of VMMC steps. About 100 steps are sufficient for orientational decorrelation of the shortest chains, while longer chains decorrelate in fewer steps, because they are perturbed more often during a step.

The initial configurations, which consisted of parallel DNA chains, were let to equilibrate for at least 2000 VMMC steps, with a large safety margin as confirmed by **Figure** **9**. The VMMC trajectories for fluctuation analysis were collected over at least 18 000 VMMC steps. In some cases, more steps were needed to obtain adequate statistics of the density fluctuation – up to 360 000 steps for the shortest chains of 16 base pairs. Standard deviations of the measured fluctuation amplitudes were estimated by block averaging, using a block of size 100.

The fluctuations Equations (13)–(17) of Fourier components of any pair of variables, denoted here as $\delta a\left(q\right)=\sum_{s}a_{s}e^{-iq\cdot r^{s}}$ and $\delta b\left(q\right)=\sum_{s}b_{s}e^{-iq\cdot r^{s}}$, are extracted as follows

(25) $$\begin{array}{ccc} & & \frac{1}{2N_{0}}\left[\langle \delta a(q)\delta b(-q)\rangle +\langle \delta a(-q)\delta b(q)\rangle \right]\\ & & \;=\frac{1}{N_{0}}〈\left[\sum_{s}a_{s}\cos\left(q\cdot r^{s}\right)\right]\left[\sum_{s}b_{s}\cos\left(q\cdot r^{s}\right)\right]\\ & & \;+\;\left[\sum_{s}a_{s}\sin\left(q\cdot r^{s}\right)\right]\left[\sum_{s}b_{s}\sin\left(q\cdot r^{s}\right)\right]〉 \end{array}$$

where $s=1\cdots N_{0}$ runs over the segments of all chains and $r^{s}$ are their positions. For segment density fluctuations δρ, $a_{s}=1$, and for the nematic fluctuations $\rho_{0}\delta Q_{ij}$, $a_{s}=\left(3t_{i}^{s}t_{j}^{s}-\delta_{ij}\right)/2$, where in B‐form DNA the segment directions $t_{s}$ are best represented by normals to the nucleobase planes. Note that the low‐q components of the extracted discrete variables are by definition “coarse‐grained” and hence the long‐wavelength correlations Equation (25) computed from the simulation data can be directly compared to the predictions of the continuum theory Equations (13)–(17).

Since the system is isotropic, all quantities depend only on the magnitude |q|=q. This fact was utilized by averaging them over spherical shells with thickness $\Delta q∼2\pi /L_{0}$, ensuring that even the smallest shell (q→0) with q=0 excluded was populated. The isotropic symmetry is broken only for non‐scalar quantities – by the direction of q defining the *z* axis, as already in Equations (8)–(12). Thus, expressing their components in such coordinate systems that $q=q{\hat{e}}_{z}$ for each q and, arbitrarily in the *xy* plane,

(26) $${\hat{e}}_{x}=\frac{{\hat{e}}_{x^{\prime }}-\left({\hat{e}}_{x^{\prime }}\cdot {\hat{e}}_{z}\right){\hat{e}}_{z}}{|{\hat{e}}_{x^{\prime }}-\left({\hat{e}}_{x^{\prime }}\cdot {\hat{e}}_{z}\right){\hat{e}}_{z}|},\;{\hat{e}}_{y}={\hat{e}}_{z}\times {\hat{e}}_{x}$$

where ${\hat{e}}_{x^{\prime }}$ is aligned with the simulation box, the components are independent of the direction of q and can be averaged. With that, for the component $\rho_{0}\delta Q_{zz}$, $a_{s}$ is $a_{s}=\left[3{\left(t^{s}\cdot {\hat{e}}_{z}\right)}^{2}-1\right]/2$ and for the components $\rho_{0}\delta Q_{\{x,y\}z}$, $a_{s}$ is $a_{s}=3\left(t^{s}\cdot {\hat{e}}_{\left\{x,y\right\}}\right)\left(t^{s}\cdot {\hat{e}}_{z}\right)/2$.

## Conflict of Interest

The authors declare no conflict of interest.

## Data Availability

The data that support the findings of this study are available from the corresponding author upon reasonable request.
